# The Diagnostic Power of Circulating miR-1246 in Screening Cancer: An Updated Meta-analysis

**DOI:** 10.1155/2023/8379231

**Published:** 2023-04-20

**Authors:** Khanh Quang Huynh, Anh Tuan Le, Thang Thanh Phan, Toan Trong Ho, Suong Phuoc Pho, Hang Thuy Nguyen, Binh Thanh Le, Thuc Tri Nguyen, Son Truong Nguyen

**Affiliations:** ^1^The Breast Unit, Cancer Center, Cho Ray Hospital, HCMC 700000, Vietnam; ^2^Department of Chemo-Radiotherapy, Cancer Center, Cho Ray Hospital, HCMC 700000, Vietnam; ^3^The Laboratory D Unit, Cancer Center, Cho Ray Hospital, HCMC 700000, Vietnam; ^4^Department of Clinical Pathology, Cho Ray Hospital, HCMC 700000, Vietnam; ^5^Department of General Director, Cho Ray Hospital, HCMC 700000, Vietnam

## Abstract

**Background:**

MicroRNA-1246 (miR-1246), an oncomiR that regulates the expression of multiple cancer-related genes, has been attracted and studied as a promising indicator of various tumors. However, diverse conclusions on diagnostic accuracy have been shown due to the small sample size and limited studies included. This meta-analysis is aimed at systematically assessing the performance of extracellular circulating miR-1246 in screening common cancers.

**Methods:**

We searched the PubMed/MEDLINE, Web of Science, Cochrane Library, and Google Scholar databases for relevant studies until November 28, 2022. Then, the summary receiver operating characteristic (SROC) curves were drawn and calculated area under the curve (AUC), diagnostic odds ratio (DOR), sensitivity, and specificity values of circulating miR-1246 in the cancer surveillance.

**Results:**

After selection and quality assessment, 29 eligible studies with 5914 samples (3232 cases and 2682 controls) enrolled in the final analysis. The pooled AUC, DOR, sensitivity, and specificity of circulating miR-1246 in screening cancers were 0.885 (95% confidence interval (CI): 0.827-0.892), 27.7 (95% CI: 17.1-45.0), 84.2% (95% CI: 79.4-88.1), and 85.3% (95% CI: 80.5-89.2), respectively. Among cancer types, superior performance was noted for breast cancer (AUC = 0.950, DOR = 98.5) compared to colorectal cancer (AUC = 0.905, DOR = 47.6), esophageal squamous cell carcinoma (AUC = 0.757, DOR = 8.0), hepatocellular carcinoma (AUC = 0.872, DOR = 18.6), pancreatic cancer (AUC = 0.767, DOR = 12.3), and others (AUC = 0.887, DOR = 27.5, *P* = 0.007). No significant publication bias in DOR was observed in the meta-analysis (funnel plot asymmetry test with *P* = 0.652; skewness value = 0.672, *P* = 0.071).

**Conclusion:**

Extracellular circulating miR-1246 may serve as a reliable biomarker with good sensitivity and specificity in screening cancers, especially breast cancer.

## 1. Introduction

Despite improvements in diagnosis and treatment, cancer is still burdened disease globally with the increased new cases and deaths over the years [1, 2]. Annual screening and earlier detection are crucial strategies that help to reduce cancer incidence and mortality [3–7]. Moreover, early detection of cancers leads to the use of less-aggressive interventions that improve patients' quality of life. Many tools have been used frequently in the surveillance of cancers as low-dose computed tomography, mammography, endoscopy, ultrasound, and serum protein markers such as carbohydrate antigen 125, 15-3, 19-9, CYFRA 21-1, carcinoembryonic antigen, squamous cell carcinoma antigen, alpha-fetoprotein, and prostate-specific antigen. Nevertheless, just a few tests have been well-accepted due to their disadvantages of expensive, invasiveness, discomfort, poor sensitivity, specificity, and a certain false-positive and false-negative rate [3, 7–9].

In recent years, liquid biopsy materials, including microRNAs (miR-21, miR-155, miR-486, etc.) in the blood and body fluids, have been attracted and extensively studied as potential biomarkers for cancer diagnosis and prognosis [10]. These are endogenous small noncoding RNAs (19-22 nt) dysregulated in cancer cells. After production, they regulate the translation of target mRNAs or can be released into circulation, then communicate and affect distant cells and tissues, leading to condition changes of tumorigenesis, angiogenesis, invasion, migration, and metastasis [10]. Among microRNAs, miR-1246 plays as an oncogenic molecule that modulates the expression of multiple genes and pathways in various cancers [11]. Previous studies presented an elevated level of miR-1246 in the blood of cancer patients compared to healthy individuals exploring its diagnostic role [12]. However, divergent conclusions on diagnostic accuracy have been shown due to the small sample size and limited cancer types [12, 13]. We aim to systematically assess the performance of extracellular circulating miR-1246 in cancer screening on a larger sample.

## 2. Materials and Methods

This meta-analysis was conducted according to the guideline of Preferred Reporting Items for Systematic Reviews and Meta-Analyses (PRISMA) [14].

### 2.1. Database Searching and Selection of Study

**Figure 1:**
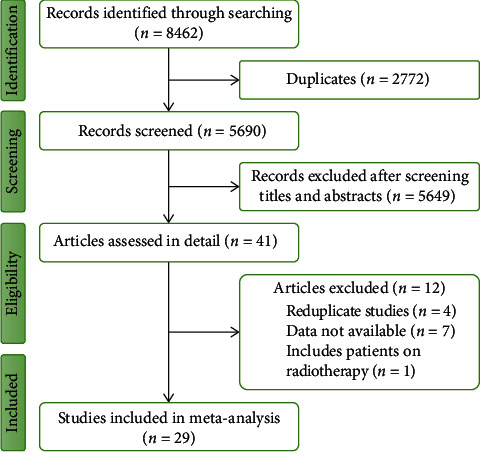
Database searching and study selection.

We searched electronic databases of PubMed/MEDLINE, Web of Science, Cochrane Library, and Google Scholar for relevant studies up to 28 November 2022. The keywords used in searching were “miR-1246,” “miR1246,” “miRNA-1246,” “miRNA1246,” “microRNA-1246,” and “microRNA1246.” Also, we reviewed citation reports of potential studies to find additional articles. After searching, all relevant studies were saved as an EndNote list. By removing duplicates (2772 records), 5690 remained for later evaluations (Figure 1). Subsequently, only 41 articles progressed to the detailed assessment step after screening titles and abstracts. Four reduplicated studies, seven with unavailable data, and one included patients on radiotherapy were excluded. Finally, 29 studies were included in this meta-analysis.

### 2.2. Quality Assessment and Data Extraction

**Figure 2:**
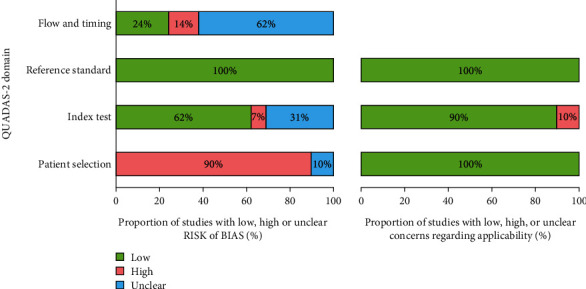
Quality of included studies regarding the risk of bias and applicability.

The quality of included studies was assessed by three independent researchers using the QUADAS-2 (Quality Assessment of Diagnostic Accuracy Studies) tool regarding the risk of bias and applicability (Figure 2) [15]. For each signaling question, “yes,” “no,” or “unclear” are phrased answers corresponding to the “low,” “high,” or “unclear” risk of bias and applicability concerns. When all signaling questions of a domain are answered “yes,” the risk of bias was judged low. If any answer “no” exists, the risk of bias was judged high. Domains were marked unclear risk of bias if any “unclear” exist without the “no” answer. In case of no consensus on judgments, three evaluators discussed in detail and determined the final decision.

Data extracted from articles include author names and country, year of publication, cancer, and control type, sample type, sample size, techniques used in experiments, and the AUC value in diagnosis. Besides, the true-positive, false-positive, true-negative, and false-negative numbers were extracted directly from articles or calculated indirectly using sensitivity and specificity corresponding to the maximum Youden's *J* index extracted from the receiving operating characteristic curve.

### 2.3. Statistical Analysis

We used the random-effects model to estimate pooled DOR, sensitivity, specificity, positive likelihood ratios, and negative likelihood ratios of circulating miR-1246 in cancer screening. Also, we constructed SROC curves and calculated summary AUC values, then compared them between groups using the bootstrap test (*B* = 2000 resampling iterations). The heterogeneity of diagnostic test accuracy between studies was measured by Higgins and Thompson's *I*^2^-statistic, which is significant if *I*^2^ ≥ 50%. Subsequently, the Leave-One-Out analysis was used to detect outlier studies, while meta-regression was performed to explore heterogeneity sources. Moreover, we used the funnel plot asymmetry statistic and the skewness of the standardized deviates to assess publication bias. All data analyses were done with the guidance of Shim et al., Noma et al., and Harrer et al. [16–18], using R statistical software v.4.2.2 (R foundation, 1020 Vienna, Austria) and packages meta, mada, metafor, dmetar, dmetatools, and altmeta. *P* < 0.05 was considered statistically significant.

## 3. Results

### 3.1. Study Characteristics

Among 29 included studies [19–47], seven studies demonstrated the diagnostic performance of circulating miR-1246 in breast cancer [21, 24, 26, 29, 41, 42, 44], while four studies showed data for colorectal cancer [20, 33, 37, 46], four others for hepatocellular carcinoma [23, 31, 32, 43], and three for esophageal squamous cell carcinoma or pancreatic cancer [19, 27, 35, 36, 39, 45] (Table 1). Twenty-six out of 29 studies included healthy individuals as the control group, which did not avoid a case-control design and thus might introduce biases according to the QUADAS-2 revised tool (Figure 2). Most studies detected miR-1246 in serum or plasma samples using the reverse transcriptase quantitative polymerase chain reaction (RT-qPCR) method. The total samples included in the meta-analysis were 5914, including 3232 cases and 2682 controls.

### 3.2. Performance of Circulating miR-1246 in Screening Cancers

**Figure 3:**
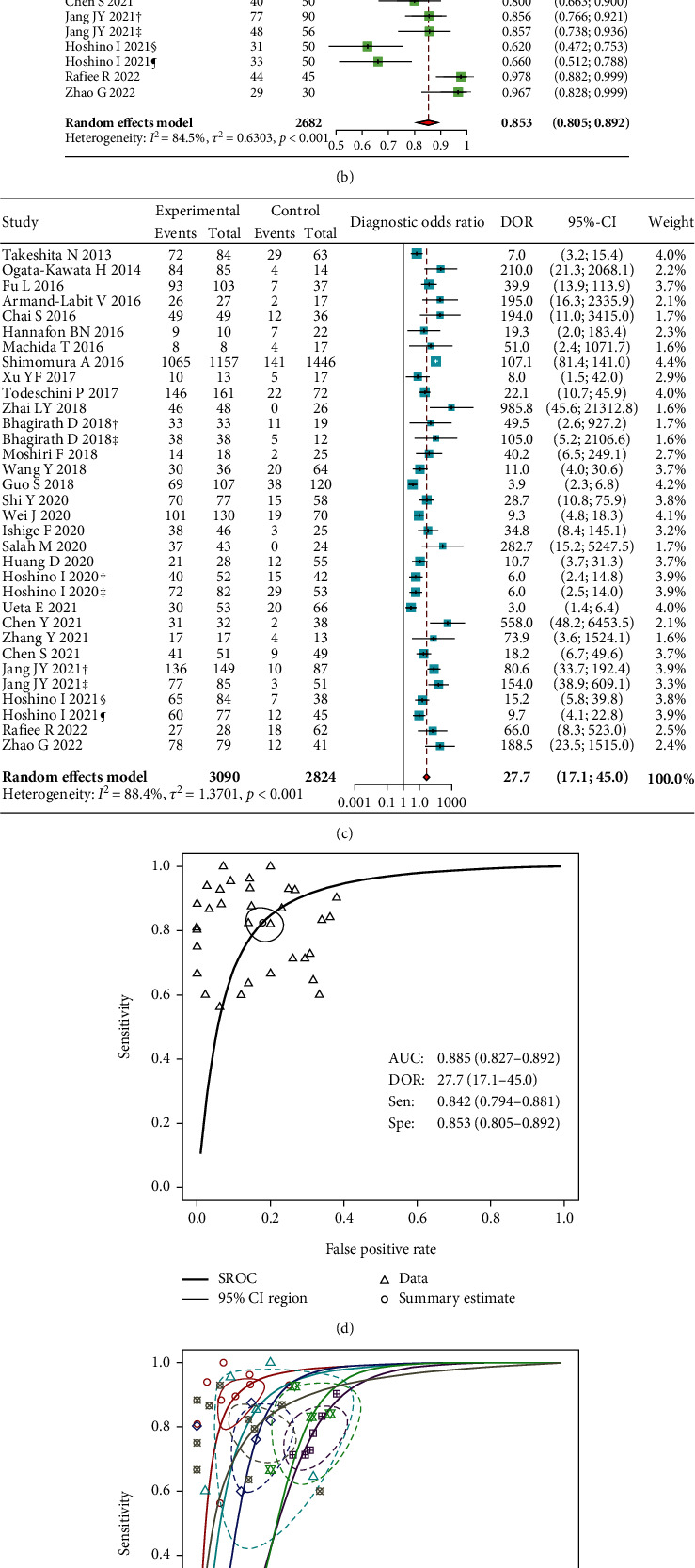
Forest plots of sensitivity (a), specificity (b), DOR (c), SROC curves (d, e), and Fagan's nomogram (f) of circulating miR-1246 in screening cancers.

The analyzed results indicated that circulating miR-1246 can differentiate cancers with 84.2% sensitivity (95% CI: 79.4-88.1) and 85.3% specificity (95% CI: 80.5-89.2, Figures 3(a) and 3(b)). Besides, the diagnostic odds ratio pooled from 29 studies was 27.7 (95% CI: 17.1-45.0, Figure 3(c)). However, heterogeneity in these analyses was substantial (*I*^2^ were 82.8%, 84.5%, and 88.4%, *P* <0.001, respectively). That is why we applied the random-effects model for the analyses.

The SROC curve of included studies shows an AUC of 0.885 (95% CI: 0.827-0.892, Figure 3(d)), suggesting that circulating miR-1246 has high diagnostic power. Remarkably, excellent performance was noted for breast cancer (AUC = 0.950, 95% CI: 0.872-0.958) compared to other types (*P* = 0.007, Figure 3(e)). With the assumed probability of suffering cancer of 55%, positive result increases the posttest possibility to 88%, while negative result drops that measure to 18% (Figure 3(f)). The positive and negative likelihood ratios were 6.35 and 0.18, respectively.

**Figure 4:**
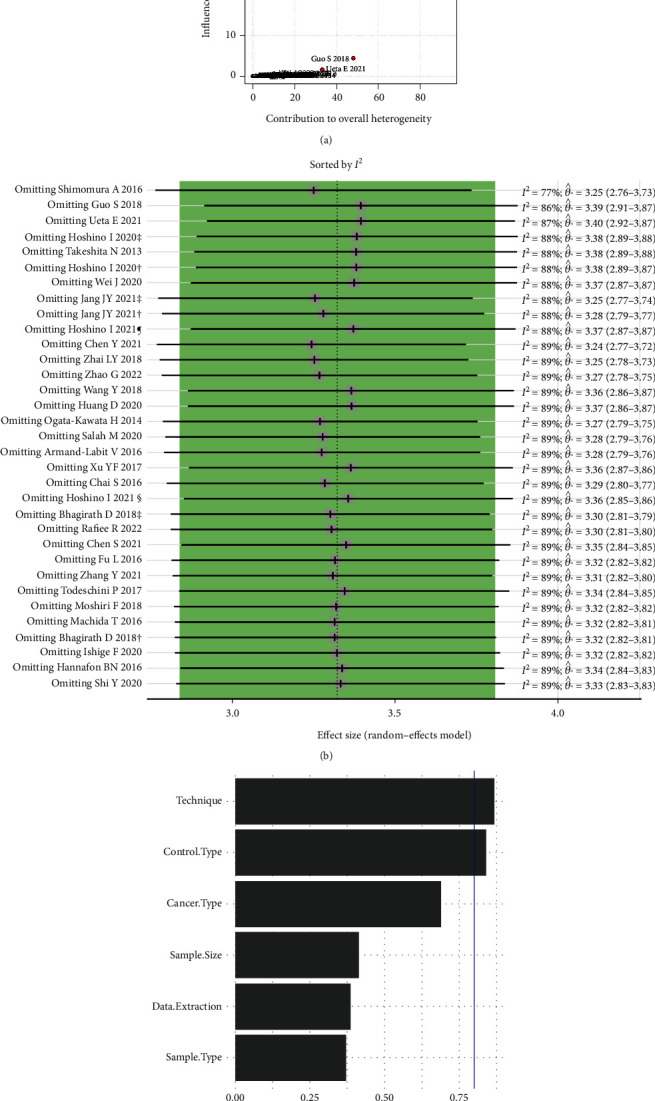
Baujat plot (a) and Leave-One-Out meta-analysis (b) for detecting outliers and important predictors for heterogeneity in DOR (c).

Because of significant heterogeneity, we performed the influence analysis and detected three outliers that contributed most to overall heterogeneity (Figures 4(a) and 4(b)). However, the heterogeneity remained high after removing these three outliers (DOR = 29.6, *I*^2^ = 67.5%, 95% CI: 52.5-77.7%, *P* < 0.001). We performed subgroup analyses and observed that cancer type, control type, sample type, sample size, technique used, and data extraction method could contribute to the sensitivity, specificity, and DOR differences between studies (Table 2). The multimodel inference analysis showed that three predictors, including technique, control type, and cancer type, are the most important ones contributing to heterogeneity overall (Akaike's information criterion was the smallest value = 102.4, Figure 4(c)). We fitted these three predictors in a meta-regression and noted that this model could explain *R*^2^ = 62.8% of the heterogeneity in DOR, and ESCC cancer type (coefficient = −1.825, *P* = 0.002), healthy control type (coefficient = 1.523, *P* = 0.015), and RT-qPCR technique (coefficient = −1.528, *P* = 0.012) are independent sources (Table 3).

**Figure 5:**
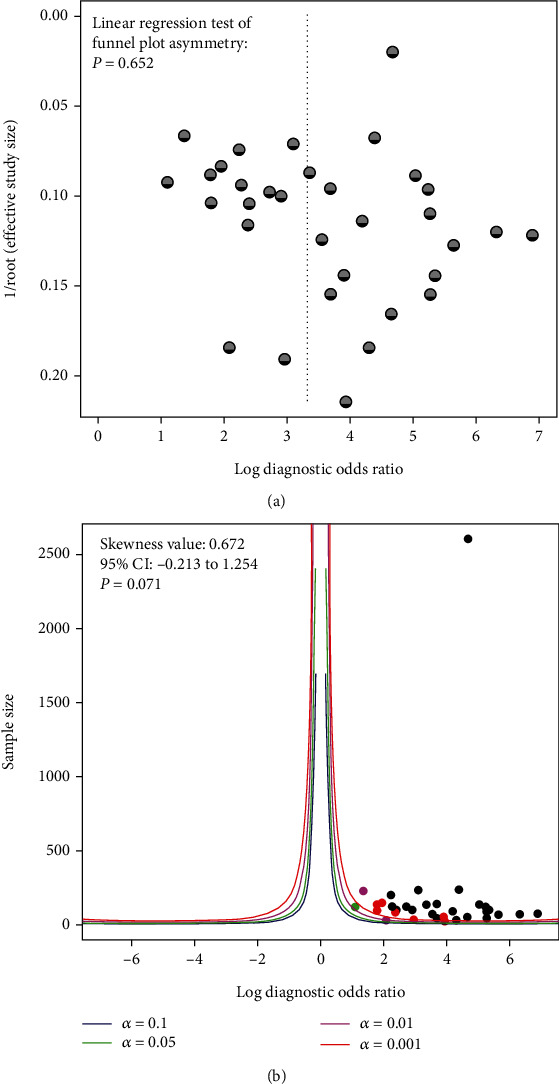
The potential of publication bias in DOR: linear regression test for funnel plot asymmetry (a) and skewness value based on the resampling method (b).

The funnel plot asymmetry test with linear regression indicated a nonsignificant publication bias in the meta-analysis (*P* = 0.652, Figure 5(a)). That is comparable with the analysis of skewness of the standardized deviates (skewness value = 0.672) (95% CI: -0.213 to 1.254, *P* = 0.071, Figure 5(b)), suggesting a low potential of publication bias [48].

## 4. Discussion

miR-1246 has been evidenced as an oncogene that regulates multiple genes (*CCNG2*, *GSK3β*, *RORα*, *AXIN2*, *DYRK1A*, *Caspase-9*, *FOXA2*, *PDGFRβ*, *p53*, *NFIB*, etc.) and signaling pathways (RAF/MEK/ERK, Wnt/*β*-catenin, NF-*κ*B, STAT3, THBS2/MMP, NOTCH2, etc.) related to the cell proliferation, angiogenesis, antiapoptosis, carcinogenesis, invasion, migration, metastasis, and therapy resistance [11]. Accordingly, recent studies indicated it as a potential biomarker for malignant tumors, but a small sample size resulted in the lack of consistent conclusions [12, 13]. The study of Wei (on 242 cases of colorectal cancer, pancreatic adenocarcinoma, and pancreatobiliary tract cancer from three original reports) exhibited an excellent efficiency of exosome miR-1246 (AUC = 0.969, 92% sensitivity, and 95.8% specificity) [12], whereas in analyses of Xie (conducted on seven individual studies, 975 cases from five cancer types including hepatocellular carcinoma, breast, colorectal, ovarian, and esophageal cancers), authors concluded that miR-1246 is a good indicator with moderate diagnostic accuracy (AUC = 0.83, 80% sensitivity, and 77% specificity) [13].

We conducted a systematic review and performed a meta-analysis on 29 individual studies from 9 countries, including 12 cancer types, over 5900 samples, and confirmed that extracellular circulating miR-1246 has good sensitivity, specificity, and robust performance in screening cancers (Figure 3(d)). Impressively, the diagnostic capacity of miR-1246 is excellent for breast cancer (Figure 3(e), Table 2). These results indicate a superior performance of circulating miR-1246 compared to the combined model of currently used tumor biomarkers [8]. In clinical practice, it is simple to integrate the miR-1246 test into the health examination program without additional blood tubes, thanks to using a small sample volume. Also, it is quantified easily by the RT-qPCR, which is currently the widely used method with a fast turnaround time. Moreover, it is a lower cost and less invasive compared to low-dose computed tomography and endoscopy tests.

This study highlights the diagnostic power of extracellular circulating miR-1246 for cancers. However, most included studies comprise healthy individuals as the control group (Table 1), which is quite different from cancerous, which thus might affect the overall results. Therefore, further clinical trial studies with cancer/benign models and early-stage diseases should be done to confirm the diagnosis role of circulating miR-1246. Another limitation of this study is the existence of significant heterogeneity that requires a cautious use of analyzed results.

## 5. Conclusion

The results of this study indicated that extracellular circulating miR-1246 has good sensitivity, specificity, and robust performance, which might serve as a reliable biomarker in screening cancers, especially breast cancer.

## Figures and Tables

**Table 1:** Characteristics of included studies.

Author	Year	Country	Case vs. control	Clinical stage	*N*, case/control	Sample	Technique	TP	FP	TN	FN	AUC	Ref.
Takeshita	2013	Japan	ESCC vs. HC	I-IV	101/46	Serum exosome	RT-qPCR	72	12	34	29	0.754	[19]
Ogata-Kawata	2014	Japan	CRC vs. HC	I-IV	88/11	Serum exosome	Microarray	84	1	10	4	0.948	[20]
Fu	2016	China	BC vs. HC	I-IV	100/40	Serum	RT-qPCR	93	10	30	7	0.904	[21]
Armand-Labit	2016	France	Melanoma vs. HC	III-IV	28/16	Plasma	RT-qPCR	26	1	15	2	0.95^£^	[22]
Chai	2016	Hong Kong	HCC vs. HC	Na	61/24	Plasma	RT-qPCR	49	0	24	12	0.982^£^	[23]
Hannafon	2016	USA	BC vs. HC	0-III	16/16	Plasma exosome	RT-qPCR	9	1	15	7	0.69^£^	[24]
Machida	2016	Japan	PT vs. HC	I-IV	12/13	Saliva exosome	RT-qPCR	8	0	13	4	0.814	[25]
Shimomura	2016	Japan	BC vs. HC	0-IV	1206/1397	Serum	Microarray	1065	92	1305	141	0.91	[26]
Xu	2017	USA	PC vs. HC	I-IIA	15/15	Plasma exosome	RT-qPCR	10	3	12	5	0.73^£^	[27]
Todeschini	2017	Italy	OC vs. HC	III-IV	168/65	Serum	RT-qPCR	146	15	50	22	0.893	[28]
Zhai	2018	China	BC vs. HC	Na	46/28	Plasma exosome	Au nanoflare probe	46	2	26	0	0.982	[29]
Bhagirath	2018^†^	USA	PCa vs. HC	IV	44/8	Serum exosome	RT-qPCR	33	0	8	11	0.926	[30]
Bhagirath	2018^‡^	USA	PCa vs. HC	IV	43/7	Serum exosome	RT-qPCR	38	0	7	5	0.933	[30]
Moshiri	2018	Italy	HCC vs. cirrhosis	Na	16/27	Plasma	ddPCR	14	4	23	2	0.97	[31]
Wang	2018	China	HCC vs. HC	I-IV	50/50	Serum exosome	RT-qPCR	30	6	44	20	0.825^£^	[32]
Guo	2018	China	CRC vs. HC	0-IV	107/120	Serum	RT-qPCR	69	38	82	38	0.681	[33]
Shi	2020	China	GC vs. HC	I-IV	85/50	Serum exosome	RT-qPCR	70	7	43	15	0.911	[34]
Wei	2020	China	PC vs. benign+HC	I-IV	120/80	Serum	RT-qPCR	101	29	51	19	0.81	[35]
Ishige	2020	Japan	PC vs. HC	0-IV	41/30	Serum	RT-qPCR	38	8	22	3	0.87	[36]
Salah	2020	Egypt	CRC vs. HC	II-III	37/30	Serum	RT-qPCR	37	6	24	0	0.924	[37]
Huang	2020	China	NSCLC vs. HC	I	33/50	Serum	RT-qPCR	21	7	43	12	0.827^£^	[38]
Hoshino	2020^†^	Japan	ESCC vs. HC	I-IV	55/39	Serum	RT-qPCR	40	12	27	15	0.816	[39]
Hoshino	2020^‡^	Japan	ESCC vs. HC	I-IV	101/34	Serum	RT-qPCR	72	10	24	29	0.779	[39]
Ueta	2021	Japan	GBC vs. benign+HC	0-IV	50/69	Serum exosome	RT-qPCR	30	23	46	20	0.646	[40]
Chen	2021	China	BC vs. HC	Na	33/37	Plasma exosome	Molecular beacon	31	1	36	2	0.983	[41]
Zhang	2021	China	BC vs. HC	I-IV	21/9	Plasma exosome	Electrochemical biosensor	17	0	9	4	0.931^£^	[42]
Chen	2021	China	HCC vs. HC	I-IV	50/50	Serum	RT-qPCR	41	10	40	9	0.865	[43]
Jang	2021^†^	Korea	BC vs. HC	0-IV	146/90	Serum	RT-qPCR	136	13	77	10	0.955	[44]
Jang	2021^‡^	Korea	BC vs. HC	0-IV	80/56	Plasma	RT-qPCR	77	8	48	3	0.963	[44]
Hoshino	2021^§^	Japan	ESCC vs. HC	I-IV	72/50	Urine	RT-qPCR	65	19	31	7	0.823	[45]
Hoshino	2021^¶^	Japan	ESCC vs. HC	I-IV	72/50	Saliva	RT-qPCR	60	17	33	12	0.802	[45]
Rafiee	2022	Iran	CRC vs. HC	I-III	45/45	Serum	RT-qPCR	27	1	44	18	0.84^£^	[46]
Zhao	2022	China	MM vs. HC	I-III	90/30	Serum	RT-qPCR	78	1	29	12	0.952	[47]

AUC: area under the receiver operating characteristic (ROC) curve; BC: breast cancer; CRC: colorectal cancer; ESCC: esophageal squamous cell carcinoma; GBC: gallbladder cancer; GC: gastric cancer; HC: healthy control; HCC: hepatocellular carcinoma; MM: multiple myeloma; Na: not available; NSCLC: non-small-cell lung cancer; OC: ovarian cancer; PC: pancreatic cancer; PCa: prostate cancer; PT: pancreatobiliary tract cancer; ddPCR: droplet digital polymerase chain reaction; RT-qPCR: reverse transcriptase quantitative polymerase chain reaction; TP: true positive; FP: false positive; TN: true negative; FN: false negative; Ref.: reference. ^†^Test set. ^‡^Validation set. ^§^Testing in urine specimens. ^¶^Testing in saliva specimens. ^£^Sensitivity and specificity values corresponding to the maximum Youden's *J* index were extracted from the ROC curve; then, true positive, false positive, true negative, and false negative numbers were calculated.

**Table 2:** Subgroup meta-analyses for sensitivity, specificity, and DOR.

Variable	Number of study	Number of case	Sensitivity				Specificity				DOR			
			Estimates, % (95% CI)	*I* ^2^, %	*P* value^∗^	*P* value^∗∗^	Estimates, % (95% CI)	*I* ^2^, %	*P* value^∗^	*P* value^∗∗^	Estimates, % (95% CI)	*I* ^2^, %	*P* value^∗^	*P* value^∗∗^
Cancer type						0.106				<0.001				<0.001
BC	7	3321	91.8 (83.9-95.9)	70.6	0.001		90.4 (84.9-94.0)	73.5	<0.001		98.5 (72.2-134.2)	29.4	0.194	
CRC	4	483	89.5 (55.2-98.3)	86.3	<0.001		87.1 (67.4-95.7)	73.3	0.011		47.6 (5.6-401.3)	87.1	<0.001	
ESCC	3	620	78.3 (70.2-84.6)	67.1	0.016		68.0 (61.6-73.9)	0.0	0.776		8.0 (5.4-11.8)	0.0	0.588	
HCC	4	328	77.1 (65.5-85.6)	67.4	0.027		87.5 (78.1-93.3)	0.0	0.751		18.6 (9.7-35.5)	29.7	0.234	
PC	3	301	84.7 (78.6-89.3)	61.9	0.072		68.0 (59.3-75.6)	0.2	0.367		12.3 (5.6-26.9)	30.2	0.239	
Others^†^	8	861	80.3 (72.6-86.3)	74.0	<0.001		89.5 (77.9-95.3)	46.4	0.061		27.5 (10.3-73.5)	75.9	<0.001	
Control type						0.292				<0.001				0.068
HC	26	5552	84.8 (79.7-88.8)	82.9	<0.001		86.5 (81.6-90.3)	82.9	<0.001		31.5 (19.1-52.1)	87.2	<0.001	
Benign	3	362	77.8 (61.5-88.5)	83.4	0.002		68.2 (60.9-74.6)	50.8	0.131		8.6 (2.3-31.7)	77.8	0.011	
Sample type						0.479				0.049				0.008
Plasma	9	544	89.1 (77.9-94.9)	64.4	0.004		92.0 (85.8-95.7)	0.0	0.716		85.8 (30.1-244.0)	50.5	0.040	
Serum	19	5101	82.5 (76.4-87.3)	87.4	<0.001		82.7 (77.1-87.1)	88.4	<0.001		21.3 (12.2-37.3)	91.8	<0.001	
Others	2	269	85.2 (78.2-90.3)	56.0	0.103		76.8 (43.0-93.6)	0.0	0.917		12.5 (6.7-23.5)	0.0	0.520	
Sample size						0.613				0.004				0.048
≥100	14	1039	85.7 (77.0-91.5)	65.3	<0.001		79.3 (73.0-84.5)	91.9	<0.001		18.8 (10.2-34.5)	93.6	<0.001	
<100	15	4875	83.3 (77.4-87.9)	88.4	<0.001		91.9 (85.5-89.2)	23.3	0.185		50.3 (23.4-108.2)	59.2	0.001	
Technique						0.009				<0.001				<0.001
RT-qPCR	23	2995	81.7 (76.3-86.2)	79.2	<0.001		82.5 (77.1-86.9)	53.0	<0.001		19.7 (12.4-31.3)	75.9	<0.001	
Others	6	2919	92.6 (86.1-96.2)	16.2	0.310		93.4 (92.0-94.5)	0.0	0.598		109.5 (83.9-142.9)	4.3	0.389	
Data extraction						<0.001				0.016				0.675
Direct	21	5420	86.9 (82.2-90.5)	82.1	<0.001		82.2 (76.7-86.6)	87.9	<0.001		28.6 (16.1-50.8)	90.8	<0.001	
Indirect	8	494	71.2 (61.4-79.4)	55.7	0.027		92.6 (85.7-96.3)	0.0	0.642		23.1 (10.2-52.3)	37.6	0.129	

BC: breast cancer; CRC: colorectal cancer; DOR: diagnostic odds ratio; ESCC: esophageal squamous cell carcinoma; HC: healthy control; HCC: hepatocellular carcinoma; PC: pancreatic cancer; PCa: prostate cancer; RT-qPCR: reverse transcriptase quantitative polymerase chain reaction; 95% CI: 95% confidence interval. ^∗^Significance for heterogeneity; ^∗∗^significance between subgroups; ^†^including gallbladder cancer (*n* = 1), gastric cancer (*n* = 1), melanoma (*n* = 1), multiple myeloma (*n* = 1), non-small-cell lung cancer (*n* = 1), ovarian cancer (*n* = 1), prostate cancer (*n* = 1), and pancreatobiliary tract cancer (*n* = 1).

**Table 3:** Meta-regression analysis for the potential sources of heterogeneity in DOR.

Predictor		Coefficient	Standard error	*P* value
Cancer type:	CRC	-1.121	0.688	0.103
	ESCC	-**1.825**	**0.587**	**0.002**
	HCC	-0.797	0.671	0.235
	PC	-0.632	0.788	0.423
	Others	-0.543	0.601	0.366

Control type:	HC	**1.523**	**0.629**	**0.015**

Technique:	RT-qPCR	**-1.528**	**0.612**	**0.012**

CRC: colorectal cancer; DOR: diagnostic odds ratio; ESCC: esophageal squamous cell carcinoma; HC: healthy control; HCC: hepatocellular carcinoma; PC: pancreatic cancer; RT-qPCR: reverse transcriptase quantitative polymerase chain reaction.

## Data Availability

All data generated or analyzed during this study are included in this published article.

## Abbreviations

AUC: Area under the receiver operating characteristic (ROC) curve
BC: Breast cancer
CRC: Colorectal cancer
ESCC: Esophageal squamous cell carcinoma
GBC: Gallbladder cancer
GC: Gastric cancer
HC: Healthy control
HCC: Hepatocellular carcinoma
MM: Multiple myeloma
Na: Not available
NSCLC: Non-small-cell lung cancer
OC: Ovarian cancer
PC: Pancreatic cancer
PCa: Prostate cancer
PT: Pancreatobiliary tract cancer
ddPCR: Droplet digital polymerase chain reaction
RT-qPCR: Reverse transcriptase quantitative polymerase chain reaction
TP: True positive
FP: False positive
TN: True negative
FN: False negative
Ref.: Reference.

## References

[1] Bray F., Ferlay J., Soerjomataram I., Siegel R. L., Torre L. A., Jemal A. (2018). Global cancer statistics 2018: GLOBOCAN estimates of incidence and mortality worldwide for 36 cancers in 185 countries. *CA: A Cancer Journal for Clinicians*.

[2] Sung H., Ferlay J., Siegel R. L. (2021). Global cancer statistics 2020: GLOBOCAN estimates of incidence and mortality worldwide for 36 cancers in 185 countries. *CA: A Cancer Journal for Clinicians*.

[3] de Koning H. J., van der Aalst C. M., de Jong P. A. (2020). Reduced lung-cancer mortality with volume CT screening in a randomized trial. *The New England Journal of Medicine*.

[4] Zielonke N., Gini A., Jansen E. E. L. (2020). Evidence for reducing cancer-specific mortality due to screening for breast cancer in Europe: a systematic review. *European Journal of Cancer*.

[5] Zhang J., Chen G., Li Z. (2020). Colonoscopic screening is associated with reduced colorectal cancer incidence and mortality: a systematic review and meta-analysis. *Journal of Cancer*.

[6] Shim J. J., Kim G. A., Oh C. H. (2020). Reduced liver cancer mortality with regular clinic follow-up among patients with chronic hepatitis B: a nationwide cohort study. *Cancer Medicine*.

[7] Fenton J. J., Weyrich M. S., Durbin S., Liu Y., Bang H., Melnikow J. (2018). Prostate-specific antigen-based screening for prostate cancer. *JAMA*.

[8] Wang H. Y., Chen C. H., Shi S. (2020). Improving multi-tumor biomarker health check-up tests with machine learning algorithms. *Cancers*.

[9] World Health Organization. Regional Office for Europe (2022). *A Short Guide to Cancer Screening: Increase Effectiveness, Maximize Benefits and Minimize Harm*.

[10] Cui M., Wang H., Yao X. (2019). Circulating microRNAs in cancer: potential and challenge. *Frontiers in Genetics*.

[11] Ghafouri-Fard S., Khoshbakht T., Hussen B. M., Taheri M., Samadian M. (2022). A review on the role of miR-1246 in the pathoetiology of different cancers. *Frontiers in Molecular Biosciences*.

[12] Wei C., Li Y., Huang K., Li G., He M. (2018). Exosomal miR-1246 in body fluids is a potential biomarker for gastrointestinal cancer. *Biomarkers in Medicine*.

[13] Xie C., Huang T., Teng Z. (2019). A meta-analysis of the diagnostic value of microRNA-1246 for malignant tumors. *Medicine*.

[14] Page M. J., McKenzie J. E., Bossuyt P. M. (2021). The PRISMA 2020 statement: an updated guideline for reporting systematic reviews. *PLoS Medicine*.

[15] Whiting P. F., Rutjes A. W., Westwood M. E. (2011). QUADAS-2: a revised tool for the quality assessment of diagnostic accuracy studies. *Annals of Internal Medicine*.

[16] Shim S. R., Kim S. J., Lee J. (2019). Diagnostic test accuracy: application and practice using R software. *Epidemiology and Health*.

[17] Noma H., Matsushima Y., Ishii R. (2021). Confidence interval for the AUC of SROC curve and some related methods using bootstrap for meta-analysis of diagnostic accuracy studies. *Communications in Statistics: Case Studies, Data Analysis and Applications*.

[18] Harrer M., Cuijpers P., Furukawa T. A., Ebert D. D. (2021). *Doing Meta-Analysis with R: A Hands-on Guide*.

[19] Takeshita N., Hoshino I., Mori M. (2013). Serum microRNA expression profile: miR-1246 as a novel diagnostic and prognostic biomarker for oesophageal squamous cell carcinoma. *British Journal of Cancer*.

[20] Ogata-Kawata H., Izumiya M., Kurioka D. (2014). Circulating exosomal microRNAs as biomarkers of colon cancer. *PLoS One*.

[21] Fu L., Li Z., Zhu J. (2016). Serum expression levels of microRNA-382-3p, -598-3p, -1246 and -184 in breast cancer patients. *Oncology Letters*.

[22] Armand-Labit V., Meyer N., Casanova A. (2016). Identification of a circulating microRNA profile as a biomarker of metastatic cutaneous melanoma. *Acta Dermato-Venereologica*.

[23] Chai S., Ng K. Y., Tong M. (2016). Octamer 4/microRNA-1246 signaling axis drives Wnt/*β*-catenin activation in liver cancer stem cells. *Hepatology*.

[24] Hannafon B. N., Trigoso Y. D., Calloway C. L. (2016). Plasma exosome microRNAs are indicative of breast cancer. *Breast Cancer Research*.

[25] Machida T., Tomofuji T., Maruyama T. (2016). MiR-1246 and miR-4644 in salivary exosome as potential biomarkers for pancreatobiliary tract cancer. *Oncology Reports*.

[26] Shimomura A., Shiino S., Kawauchi J. (2016). Novel combination of serum microRNA for detecting breast cancer in the early stage. *Cancer Science*.

[27] Xu Y. F., Hannafon B. N., Zhao Y. D., Postier R. G., Ding W. Q. (2017). Plasma exosome miR-196a and miR-1246 are potential indicators of localized pancreatic cancer. *Oncotarget*.

[28] Todeschini P., Salviato E., Paracchini L. (2017). Circulating miRNA landscape identifies miR-1246 as promising diagnostic biomarker in high-grade serous ovarian carcinoma: a validation across two independent cohorts. *Cancer Letters*.

[29] Zhai L. Y., Li M. X., Pan W. L. (2018). In situ detection of plasma exosomal microRNA-1246 for breast cancer diagnostics by a Au nanoflare probe. *ACS Applied Materials & Interfaces*.

[30] Bhagirath D., Yang T. L., Bucay N. (2018). MicroRNA-1246 is an exosomal biomarker for aggressive prostate cancer. *Cancer Research*.

[31] Moshiri F., Salvi A., Gramantieri L. (2018). Circulating miR-106b-3p, miR-101-3p and miR-1246 as diagnostic biomarkers of hepatocellular carcinoma. *Oncotarget*.

[32] Wang Y., Zhang C., Zhang P. (2018). Serum exosomal microRNAs combined with alpha-fetoprotein as diagnostic markers of hepatocellular carcinoma. *Cancer Medicine*.

[33] Guo S., Zhang J., Wang B. (2018). A 5-serum miRNA panel for the early detection of colorectal cancer. *Oncotargets and Therapy*.

[34] Shi Y., Wang Z., Zhu X. (2020). Exosomal miR-1246 in serum as a potential biomarker for early diagnosis of gastric cancer. *International Journal of Clinical Oncology*.

[35] Wei J., Yang L., Wu Y. N., Xu J. (2020). Serum miR-1290 and miR-1246 as potential diagnostic biomarkers of human pancreatic cancer. *Journal of Cancer*.

[36] Ishige F., Hoshino I., Iwatate Y. (2020). MIR1246 in body fluids as a biomarker for pancreatic cancer. *Scientific Reports*.

[37] Salah M., Shaheen I., El-Shanawany P. (2020). Detection of miR-1246, miR-23a and miR-451 in sera of colorectal carcinoma patients: a case-control study in Cairo university hospital. *African Health Sciences*.

[38] Huang D., Qu D. (2020). Early diagnostic and prognostic value of serum exosomal miR-1246 in non-small cell lung cancer. *International Journal of Clinical and Experimental Pathology*.

[39] Hoshino I., Ishige F., Iwatate Y. (2020). Usefulness of serum miR-1246/miR-106b ratio in patients with esophageal squamous cell carcinoma. *Oncology Letters*.

[40] Ueta E., Tsutsumi K., Kato H. (2021). Extracellular vesicle-shuttled miRNAs as a diagnostic and prognostic biomarker and their potential roles in gallbladder cancer patients. *Scientific Reports*.

[41] Chen Y., Zhai L. Y., Zhang L. M. (2021). Breast cancer plasma biopsy by in situ determination of exosomal microRNA-1246 with a molecular beacon. *Analyst*.

[42] Zhang Y., Zhang X., Situ B. (2021). Rapid electrochemical biosensor for sensitive profiling of exosomal microRNA based on multifunctional DNA tetrahedron assisted catalytic hairpin assembly. *Biosensors and Bioelectronics*.

[43] Chen S., Fu Z., Wen S. (2021). Expression and diagnostic value of miR-497 and miR-1246 in hepatocellular carcinoma. *Frontiers in Genetics*.

[44] Jang J. Y., Kim Y. S., Kang K. N., Kim K. H., Park Y. J., Kim C. W. (2021). Multiple microRNAs as biomarkers for early breast cancer diagnosis. *Molecular and Clinical Oncology*.

[45] Hoshino I., Ishige F., Iwatate Y. (2021). Cell-free microRNA-1246 in different body fluids as a diagnostic biomarker for esophageal squamous cell carcinoma. *PLoS One*.

[46] Rafiee R., Razmara E., Motavaf M. (2022). Circulating serum miR-1246 and miR-1229 as diagnostic biomarkers in colorectal carcinoma. *Journal of Cancer Research and Therapeutics*.

[47] Zhao G., Jing X., Li Z., Wu X., Gao Z., Ma R. (2022). The diagnostic and prognostic values of circulating miRNA-1246 in multiple myeloma. *Hematology*.

[48] Murad M. H., Chu H., Lin L., Wang Z. (2018). The effect of publication bias magnitude and direction on the certainty in evidence. *BMJ Evidence-Based Medicine*.

